# Human infertility: are endocrine disruptors to blame?

**DOI:** 10.1530/EC-13-0036

**Published:** 2013-09-17

**Authors:** André Marques-Pinto, Davide Carvalho

**Affiliations:** 1 Serviço de EndocrinologiaFaculdade de Medicina da Universidade do PortoAl. Prof. Hernâni Monteiro4200-319, PortoPortugal; 2 Departamento de Endocrinologia, Diabetes e MetabolismoCentro Hospitalar de São JoãoPortoPortugal

**Keywords:** endocrine disruptors, reproduction, infertility, male, female

## Abstract

Over recent decades, epidemiological studies have been reporting worrisome trends in the incidence of human infertility rates. Extensive detection of industrial chemicals in human serum, seminal plasma and follicular fluid has led the scientific community to hypothesise that these compounds may disrupt hormonal homoeostasis, leading to a vast array of physiological impairments. Numerous synthetic and natural substances have endocrine-disruptive effects, acting through several mechanisms. The main route of exposure to these chemicals is the ingestion of contaminated food and water. They may disturb intrauterine development, resulting in irreversible effects and may also induce transgenerational effects. This review aims to summarise the major scientific developments on the topic of human infertility associated with exposure to endocrine disruptors (EDs), integrating epidemiological and experimental evidence. Current data suggest that environmental levels of EDs may affect the development and functioning of the reproductive system in both sexes, particularly in foetuses, causing developmental and reproductive disorders, including infertility. EDs may be blamed for the rising incidence of human reproductive disorders. This constitutes a serious public health issue that should not be overlooked. The exposure of pregnant women and infants to EDs is of great concern. Therefore, precautionary avoidance of exposure to EDs is a prudent attitude in order to protect humans and wildlife from permanent harmful effects on fertility.

## Introduction

Infertility, which is defined as the inability to conceive after 1 year of unprotected intercourse, has a global prevalence of 9% (1). Among infertile couples, it is estimated that the cause is predominantly feminine in 38% and primarily masculine in 20%, while 27% have both male and female abnormalities, and no evident cause is identified as for the remaining 15% (2).

Since the mid-20th century, numerous studies have reported an increasing incidence of human reproductive diseases and a consequent decline in reproductive function worldwide (3). Given the short time frame, genetic changes cannot explain it. Thus, environmental substances may be accountable for the observed trends (4, 5). Indeed, both humans and wildlife are exposed to copious potentially hazardous chemicals that are released into the environment at an alarming rate (6).

One of the most significant landmarks in endocrinology over the past century was the recognition that some of these chemicals are able to disrupt the closed feedback loops of the hormonal and homeostatic systems, thus being named endocrine disruptors (EDs) (7). The group of known ED is extremely heterogeneous. It embraces ubiquitous synthetic substances used as industrial lubricants and solvents, and their by-products: polychlorinated biphenyls (PCB) (8), polybrominated diphenyl ethers (PBDE) (9) and dioxins such as 2,3,7,8-tetrachlorodibenzo-*p*-dioxin (TCDD) (10); plastics: bisphenol A (BPA) (11) and bisphenol S (BPS) (12); plasticisers: phthalates (13); pesticides: atrazine (14), cypermethrin (15), dichlorodiphenyltrichloroethane (DDT) (16), dieldrin (17), methoxychlor (MXC) (16) and vinclozolin (VCZ) (18); and drugs: diethylstilbestrol (DES) (19) and ethinyl oestradiol (EE) (20), as well as non-steroidal anti-inflammatory drugs (NSAID) and acetaminophen (21). Natural chemicals such as genistein, a phytoestrogen (22) and heavy metals (23) can also have endocrine-disruptive effects.

Consistent detection of ED residues in human serum, seminal plasma and follicular fluid has raised concern that environmental exposure to ED is affecting human fertility (24). Though ED are not considered major teratogens, reproductive function – from gamete production through to intrauterine development of the offspring – is believed to be particularly susceptible to endocrine disruption, triggering morphological and functional abnormalities (25, 26, 27).

The main purpose of this paper is to review and summarise the major scientific developments on the topic of human infertility associated with ED exposure, integrating evidence from epidemiological and experimental studies. Examples of well-known and hypothetical ED are selected to highlight the potential effects of ED on human fertility, identifying future research directions.

## Methods

The PubMed database was used to search for articles published up to 31st May 2013, using the following MeSH keywords: endocrine disruptors, fertility and infertility. Only studies using the English language were considered. Altogether, 368 papers were retrieved. The abstract of every article was read. The leading review criterion was human epidemiological studies in which a link between ED exposure and infertility was evaluated. Moreover, as the interpretation of the scarce epidemiological data may be biased by many confounding factors, supporting experimental research in animal models was also considered. Although there has been an effort to list and rank all possible ED (28, 29), the number of evaluated chemicals remains limited. The full texts of 225 selected articles were retrieved and read. Furthermore, the bibliographies from 41 selected review articles were analysed, and 153 further papers were read. Overall, 198 articles were deemed relevant and included in this review.

## Endocrine disruptors

### Mechanisms of action

Given the complexity of the endocrine system, the mechanisms of action of ED are difficult to unravel. So far, most EDs are known to act as imperfect ligands (either agonists or antagonists) to nuclear and membrane receptors (for both steroidal and non-steroidal hormones, and also for orphan receptors), thus interfering with hormone-regulated cell signalling pathways and gene expression (30). The relative importance of these types of receptors on the magnitude of the effects of ED remains unclear. Of note, while exogenous hormonally active agents are considered harmful in healthy individuals, they are the basis for hormonal therapy in some endocrinological diseases and hormone-dependent cancers (31). Thus, in those circumstances, they are not considered ED.

Most EDs are supposed to act through several mechanisms, which may have synergistic or antagonistic outcomes (32). Many are substances with oestrogenic/anti-androgenic activity that act by interfering with the oestrogen receptors (ER) or the androgen receptor (AR) (see Table 1).

Apart from ER and AR, the aryl hydrocarbon receptor (AhR) is the protein most studied regarding its interaction with ED. This orphan receptor acts as a transcription factor for detoxifying enzymes (43). Dioxins and some PCB exert their endocrine-disruptive effects through binding to AhR and impairing the usual gene transcription response (44). AhR ligands enhance the degradation of sex steroid receptors (45).

Some EDs are also capable of modifying hormone bioavailability by interfering with its secretion and transport or disrupting the enzymatic pathways involved in hormone synthesis and metabolism (46, 47). For instance, in either sex, androgens give rise to oestrogens, through aromatase, so together they play a vital role in homoeostasis (48, 49). EDs that interfere with aromatase (BPA (50) and atrazine (51) stimulate its activity, while DDT and phthalates (47) inhibit it) disrupt the delicate androgen–oestrogen balance required for proper reproductive function. Recently, many anti-virilising EDs (e.g. phthalates and BPA) have been found to be powerful cyclooxygenase inhibitors, reducing prostaglandin synthesis, and this might be the foremost mechanism by which they exert their effects (52).

### Dose–effect curves

The principle of endocrine disruption has always been controversial: it has been difficult to determine the lowest observed adverse effect level (LOAEL) and whether it is likely to be found *in vivo* (53). Current postulated LOAEL for most ED are outdated (54). As an example, BPA has been found to induce detrimental reproductive effects in levels several-fold below its conventional LOAEL – 50 mg/kg of body weight (BW) per day (55).

Perhaps expectably, there is a sharp division between those who report detrimental effects of ED at environmental levels (micro- to picomolar range) – mostly academic experts – and those who appear unable to do so at any concentrations – industry corporations (56). Current data state that the most potent ED effects arise from minute environmental doses rather than from higher doses, which may induce receptor down-regulation and cytotoxicity (54).

Hormone-mimetic ED, similarly to endogenous hormones, may have non-monotonic tissue-specific effects due to: receptor selectivity, down-regulation/desensitisation, competition and negative feedback loops (57). EDs non-monotonic effects may also arise from the overlap of two or more monotonic responses through different pathways, resulting in biphasic or multiphasic curves (58).

Reliable evidence of both non-monotonic curves and low-dose detrimental effects has been gathered for BPA, many pesticides (54) and phthalates as well (59). Therefore, a threshold dose cannot be presumed, neither can low-dose effects be predicted from high-dose effects (30). However, assuming equivalent exposures, the incidence of detrimental reproductive effects of some ED may be significantly higher in vulnerable individuals, owing to several factors such as the genetic background, window of exposure and pre-existing disease. Nonetheless, these issues remain controversial (60).

### Human exposure

Populations are exposed to ED in air, water, food and in a variety of industrial products, including personal care goods. The mixture of ED that leaches into the soil and waterbodies (e.g. pesticides, contraceptive pills and other chemicals from urban and agricultural waste) accumulates in the environment and in animals higher up on the food chain (6, 7). Indeed, some EDs that were banned decades ago, namely DDT and PCB, are still found in human serum (24). This is due to their lipophilicity and resistance to biodegradation (61).

Although there is chronic exposure to ED through inhalation and skin contact (62), the major route of human exposure is ingestion of food (e.g. meat, fish, dairy products and vegetables), as well as plain water and other beverages. ED-contaminated food and water may contain environmental pollutants such as pesticide residues (63) and heavy metals (23), in addiction to processing aids and anabolic steroids used in food production. Most individuals have traceable amounts of these substances in their serum or urine (3, 64).

Recent studies have concluded that plastic packaging is an important source of ED in the average human diet (65). Repeated exposure of food-contact materials to u.v. light, heat and acidic/alkaline contents may cause polymers to breakdown into monomers as phthalates and BPA, which then leach into food and beverages (66). Thus, there is chronic intake of ED even from bottled water (67). Some of these EDs are being replaced by heat-stable analogues: many ‘BPA-free’ products contain BPS instead, which also exerts both genomic and non-genomic endocrine-disruptive effects at environmental concentrations as low as picomolar, leading to concerns regarding its safety (12, 38).

The average diet also contains natural ED such as phytoestrogens, which are compounds possessing strong oestrogen-like activity (22, 36). The eventual health benefits of phytoestrogens on cardiovascular and menopause-related disorders (68) and the apparent absence of major long-term adverse effects have led to an increased consumption of these substances, mainly through soy-based food (69). However, effective but harmless doses have yet to be established. Studies have revealed that infants ingesting soy-based formulas may have a phytoestrogen serum concentration 13 000–22 000 times higher than endogenous oestrogen levels (70), leading to concerns about its possible adverse effects on brain and reproductive organ morphological and functional development and, ultimately, on fertility (71).

### Windows of susceptibility

Human susceptibility to disruption during development has been proven (72, 73). Intrauterine exposure to ED may result in long-lasting changes. These may lead to immediate or deferred adverse outcomes on development and reproduction (74). The timing of exposure may explain this difference (75). If it occurs during critical windows, adverse effects may be very drastic and irreversible, including congenital abnormalities. On the contrary, if it happens during sensitive, non-critical windows, detrimental outcomes may still arise, such as mild functional deficits and adult-onset diseases.

#### Developmental programming

The prenatal period has become a significant research topic regarding ED exposure because the placenta causes accumulation of ED in the foetus (76). BPA and other ED have low binding affinity to the sex hormone-binding globulin and α-fetoprotein, which prevent maternal sex hormones from crossing the placenta (77). Furthermore, detoxifying metabolic pathways only maturate after birth (78). ED may therefore reach hormone-sensitive foetal tissues (e.g. the urogenital sinus and brain) and disrupt their proper development (see below). As programming of the hypothalamus–pituitary–gonadal (HPG) axis occurs during this period, ED exposure may determine fertility in the adulthood (79).

Epigenetic modifications may have an important role in the observed ED effects in gametogenesis and foetal development (see below). The epigenome refers to changes made in gene expression by altering DNA structure through DNA methylation and microRNA, among other mechanisms, without changing the actual genomic sequence (80). BPA, phthalates and VCZ can alter the gene expression and imprinting patterns in mouse embryos (81). Very recently, intrauterine BPA exposure at environmental doses was shown to impair steroidogenesis in sheep by down-regulating gonadal microRNA (82). These findings may partially explain the biological relevance of ED on gonadal differentiation.

#### Multi- and transgenerational effects

EDs have been shown to disrupt the development of the human reproductive system, impairing fertility not only in directly exposed offspring but also in subsequent generations. A vast array of reproductive abnormalities has been reported in the offspring of women treated with DES during the mid-20th century, for miscarriage prevention (19, 83). Recently, a French epidemiologic study has shown that the grandchildren of DES-exposed women have a higher incidence of genital malformations, which may be explained by epigenetic changes of the AR gene transmitted through the female germ line (84).

Other ED have multigenerational effects: the offspring of TCDD-exposed mice show fertility disorders up to the third generation (85); the third generation of mice exposed *in utero* to environmental levels of PCB presented morphological reproductive abnormalities and impaired gamete quality (8).

Male germ cells are considered as the most vulnerable cells, as they have distinctive methylation patterns and epigenetic markers (80). Transient developmental exposure of male rats to VCZ and MXC during the epigenetic-reprogramming stage induces poor semen quality up to the fourth generation (86).

ED exposure in pregnant females can directly cause detrimental effects in the next two generations through the foetus and its germline, which is already formed. Only adverse effects in the third generation and beyond are considered truly transgenerational, as they are transmitted solely through the germline (87).

As current assisted reproduction techniques do not necessarily address the underlying infertility problem, their escalating use may accidentally convey serious genetic and epigenetic anomalies (27).

#### Susceptible population groups

Millions of children are conceived by women while on contraceptive pills containing EE. Albeit most do not show conspicuous congenital abnormalities, long-term reproductive consequences may ensue in adulthood (88). Breastfeeding is another significant period of exposure to ED (89). As many ED accumulate in fat-rich tissues such as the breast, both mother and foetus are exposed to relatively high levels of these substances (90, 91). For these reasons, women of childbearing age, specifically those who are pregnant/breastfeeding, constitute a population of utmost importance regarding ED exposure. Likewise, newborns and children deserve special consideration, as they have proportionally higher food and water intakes than adults, leading to a potentially higher body burden of such chemicals (92).

### Effects of ED mixtures

ED may act synergistically to produce adverse effects at doses far below individual LOAEL, if there is enough overall exposure (93). Indeed, a combination of estrogenic ED at environmentally relevant doses was shown to lead to greater cellular disruption than single ED exposure (94). Furthermore, a study addressing the effects of developmental exposure of rats to a mixture of diverse-acting anti-androgenic ED has shown synergistic effects regarding the incidence of reproductive tract anomalies (95). In view of recent evidence, a number of brief intrauterine exposures to therapeutic doses of NSAID or acetaminophen (21, 96) adding to the potential long-lasting inhibition of prostaglandin synthesis by other ED could seriously impact human reproductive health by decreasing steroidogenesis.

Additionally, it is hypothesised that phytoestrogens, among other EDs, may be capable of altering cell responsiveness to endogenous hormones and other ED, thereby inducing wider negative effects when there is concomitant exposure (97). Two studies in rats have suggested that the effects of chronic ingestion of a low-dose genistein and VCZ mixture (at 1 mg/kg BW per day) diverge from those arising from exposure to each substance individually: genistein may potentiate the detrimental effects of VCZ when exposure occurs throughout adulthood (98) or ease them if exposure stops at birth (99). ED mixtures most likely produce very complex dose–response curves due to overlapping additive/synergistic effects, and may lead to more severe consequences than previously ascertained. Conversely, their effects may be antagonistic, and thus reciprocally annulled.

## ED and the male reproductive system

### Trends in semen quality

Over the last decades, epidemiological studies have reported an ominous growth in the incidence of male infertility, accompanied by decreasing sperm quality, thus reflecting impaired spermatogenesis (100). A large review of international studies showed that, over 50 years, the global average sperm count dropped by half (from 113 to 66 million/ml), reflecting an average yearly decrease of 1%, and sperm morphology/motility abnormalities significantly increased (101). A subsequent larger study confirmed the declining sperm concentration at a yearly rate of 1.5–3% (102). However, some consider those results are biased (103).

Studies comparing male reproductive disorders in the Nordic–Baltic countries have reported an East–West gradient showing higher reproductive tract abnormalities and infertility rates in Denmark compared with Finland (104, 105). ED may explain these differences because the Danish seem to have higher ED body burdens than the Finnish (90).

Actually, several epidemiological studies have found an association between inferior semen quality parameters and increased urinary and serum levels of phthalates (106), PCB (107), PBDE (108, 109) and BPA (110). ED may disrupt spermatogenesis by interfering with germ cells and spermatogenesis-supporting cells (111) (see Table 2). Interestingly, it has been shown that intrauterine exposure to BPA disrupts the blood–testis barrier, which may lead to infertility in adulthood through germ cell loss via immunological activity (79, 115).

### The testicular dysgenesis syndrome

There is an epidemiological correspondence between lower semen quality and higher incidences of cryptorchidism, hypospadias and testicular cancer (116). These disorders have been regrouped as the testicular dysgenesis syndrome (TDS) (117), as they probably arise from intrauterine disruption of proper testicular development and function (118) under ED exposure (119). Impaired Leydig cells function is the main cellular trait of TDS (120, 121). In mild cases, men have low testosterone levels, slightly decreased penile/testicular volumes and poor semen quality, while in the more severe cases there is also hypospadias or cryptorchidism and an increased risk of testicular cancer (122). ED exposure has been suggested to have triggered the escalation of milder TDS cases, and it may explain a number of idiopathic infertility cases (123), which constitute half the men presenting at infertility clinics (124).

Epidemiological data suggest that human developmental exposure to environmental levels of ED (e.g. phthalates, PCB and pesticides) is indeed connected to an increased risk of TDS features such as hypospadias and cryptorchidism (91, 125, 126, 127).

Assuming the same circumstances of exposure, deleterious effects of ED may be more severe in individuals with genetic susceptibility. There are AR and ER-α genetic polymorphisms that cause mild functional impairments (128, 129). They can be expected to bring about manifest forms of TDS, when combined with ED exposure (119). Indeed, among men exposed to PCB and DDT, those having particular AR polymorphisms were found to have significantly inferior sperm quality (130). Furthermore, a correlation has been reported between cryptorchidism and ED-vulnerable ER-α polymorphisms (131).

#### Hypospadias

Hypospadias, a condition in which the urethral meatus is on the ventral side of the penis, affects about 0.4% of males at birth and has been reported to have increased significantly over recent decades (132). EDs are regarded as a contributing factor, as VCZ (133) and phthalates (134) consistently induce hypospadias in the laboratory animals.

#### Cryptorchidism

Cryptorchidism is defined as the failure of one or both testicles to descend into the scrotal sac and is the most common congenital abnormality in male children, affecting 2–4% of full-term males (104). Epidemiological studies suggest that the incidence of cryptorchidism is rising (135). It is currently the best characterised risk factor for infertility and testicular cancer in adulthood (97).

Testicular migration is a complex process involving a transabdominal stage and a transinguinal one. Developmental exposure to ED may act on Leydig cells thus disrupting both stages by i) reducing insulin-like factor 3 expression (136) and ii) impairing steroidogenesis (resulting in relative testosterone deficiency) respectively (119). Exposure to some ED, such as PBDE, through breastfeeding has been correlated with cryptorchidism in new borns (76). In a recent epidemiological study, NSAID or acetaminophen consumption during pregnancy has been shown to be directly related to a higher risk of cryptorchidism in male infants, if intake had taken place for longer than one week or if there had been simultaneous ingestion of more than one of those drugs (21).

### The differentiation of the male reproductive system

The differentiation of the male reproductive system is entirely dependent on foetal testicular androgen production (137). Thus, disruption of androgen activity by ED during the virilisation period (around 8–14 weeks into human foetal development) will perhaps cause TDS (138). Moreover, disproportionate oestrogenic exposure at this point may disturb the delicate androgen–oestrogen balance, leading to adverse consequences (139).

A recent study including a thousand new borns has found a linear correlation between maternal exposure to ED (e.g. pesticides and phytoestrogens) and lower testosterone levels, smaller penile length and higher incidences of reproductive anomalies including hypospadias (140).

In animal models, pregnant mice orally exposed to phthalates at doses as low as 1 μg/kg BW per day consistently gave birth to male offspring presenting a syndrome of reproductive anomalies including cryptorchidism, testicular injury, reproductive tract malformations and shorter anogenital distance (AGD) (59, 134), reflecting ineffective perineal virilisation (141). This pattern of effects parallels TDS (142). Actually, developmental exposure to phthalates at environmental doses seems to cause reduced AGD in male infants (143).

Similarly to rodents, human male infants exhibit twice as long an AGD than females (144). Reduced male AGD may be considered a predictor of infertility as it correlates with poorer sperm quality parameters in otherwise normal men (145). Furthermore, hypospadias and cryptorchidism are also associated with shorter AGD (146).

Other anti-androgenic ED can induce TDS in animals: rats exposed to 150 mg/kg BW per day of acetaminophen during foetal development had AGD reductions comparable to those induced by phthalates (21). Additionally, intrauterine exposure to VCZ produces a wide spectrum of reproductive disorders (147). In a study, all male rats exposed *in utero* to 20–100 mg/kg BW per day of VCZ showed hypospadias and minute sperm counts (133).

Though average human ED exposure levels may be lower than those customarily used in animal studies, certain population clusters may be exposed to higher levels. Actually, occupational pesticide exposure has been connected to male infertility (125, 148, 149, 150).

## ED and the female reproductive system

### The ovarian dysgenesis syndrome

Data concerning ED effects on the female reproductive system and fertility are scant. Still, a correlation between developmental ED exposure and long-term effects is suggested (151). There is a significantly higher risk of infertility in women who have high serum concentration of BPA (152, 153), as well as in those whose mothers had high maternal serum concentrations of DDT during pregnancy (154). Moreover, occupational exposure to ED such as pesticides and plastics is a risk factor for female infertility (155).

The array of female reproductive disorders where ED have been implicated includes endometriosis, disorders of the uterus and disorders of the ovary, such as premature ovarian failure (POF) and polycystic ovary syndrome (PCOS) (26). The incidence of these conditions is growing (72). As they may arise from impaired ovarian development and function, the ovarian dysgenesis syndrome has recently been suggested as the female form of TDS (156).

#### Endometriosis

Endometriosis affects up to 10% of women of childbearing age, causing infertility in about half those women (157). Recently, EDs have been proposed as a possible contributing factor for its development and exacerbation (158). Indeed, a significantly higher BPA (159) and phthalate (160) serum concentration has been found in women with this condition. Furthermore, women exposed to DES *in utero* may have an 80% higher risk of endometriosis than unexposed women (161).

Experimental studies support this hypothesis, as intrauterine exposure of mice to BPA (162) or TCDD (85) produces an endometriosis-like adult uterine phenotype. A recent study has shown that women with endometriosis have significantly higher concentrations of TCDD and PCB in the peritoneal fluid (163), possibly leading to chronic inflammation, which may result in the stimulation of endometrial cells derived from retrograde menstruation (164).

#### Ovarian pathology

There are growing concerns about the reproductive outcomes of ovarian exposure to ED during foetal development and after birth (165). Female germ cells are a fixed population, unlike male germ cells. Therefore, exposure of hormone-responsive, primordial and preantral follicles to ED may impair folliculogenesis, inducing meiotic aberrations (e.g. aneuploidy and multiple oocyte follicles) or even follicular atresia (see Table 3). Ultimately, ED may lead to depletion of follicular reserves, resulting in POF (176). This is a syndrome consequent to impaired ovarian function before the age of 40 years, affecting about 1% of women (177).

Granulosa and theca cells, which are crucial for ovarian steroidogenesis and oocyte development, are also a target for ED (48). Chronic exposure to TCDD at environmental levels (lower than 1 ng/kg BW per day) induces ovarian insufficiency in rats by reducing steroidogenesis (10).

PCOS, consisting of hyperandrogenemia and chronic anovulation, affects 5–8% of women of childbearing age often leading to infertility (178). Higher serum BPA levels have been reported in women with PCOS compared with healthy women (153, 179).

### The differentiation of the female reproductive system

Proper differentiation of the female reproductive system is regulated by oestrogens, but it proceeds even in their absence – it is the default developmental pathway (180). Nevertheless, oestrogenic overstimulation is known to result in irreversible abnormalities (19, 181).

The development of the female reproductive system is regulated by the differential expression of *HOX* genes in the Müllerian duct (182). Disruption of the precise chronological regulation of *HOXA10* by ED that either up-regulate (e.g. BPA) or down-regulate (e.g. DES and MXC) its expression has been shown to lead to uterine abnormalities and infertility (183). DES has also been found to contribute to uterine abnormalities by reducing the expression of other developmental genes such as the *WNT7* or *MSX2* genes (184).

## Central actions of ED

### Regulation of gonadotropin secretion

ED may modify steroidogenesis both locally and through the HPG axis (7). The human HPG axis is active *in utero* and during the first year of life (185). Afterwards, gonadotropin secretion is reduced until puberty, when sequential endocrine changes set in motion the development of secondary sexual characteristics that will lead to sexual maturation (186).

Kisspeptin is broadly recognised as a fundamental activator of the HPG axis, at the onset of puberty (187). In rats, neonatal exposure to oestrogenic ED, such as BPA and genistein, suppresses kisspeptin synthesis (188, 189).

Some PCBs have been shown to alter gonadotropin-releasing hormone (GnRH) synthesis (190) and to decrease GnRH release (191). Conversely, DDT and BPA stimulate it (192). In rats, perinatal exposure to environmental BPA doses, below the current LOAEL, induced defective GnRH pulses up to adulthood, leading to infertility (193).

The biological ED effects through GnRH and kisspeptin neurons and the relative importance of disruption in each those cell clusters on the onset of puberty and fertility throughout life through remain unclear.

Disruption of the HPG axis leading to gonadal insufficiency by reducing steroidogenesis, following exposure to DES (113), PCB (190) and atrazine (14), was demonstrated in rats. Long-lasting reproductive disorders induced by developmental ED exposure may be more likely to arise from a dysfunctional HPG axis (194). Thus, the primary target of developmental ED exposure might be the hypothalamus and the pituitary gland rather than the gonads themselves (195).

### Sexually dimorphic neural circuitry

Sex steroids have prominent roles in the differentiation of several sexually dimorphic neural circuits (195, 196). ED may cross the immature blood–brain barrier (11) and thereby reverse the neurochemical phenotype of these areas. Actually, developmental exposure to BPA, MXC and VCZ has been shown to produce gender-inadequate adult behaviours (197), possibly by disrupting specific neural pathways (e.g. nitrergic fibres) that influence complex functions and behaviours such as those related to reproduction (198).

## Conclusion

This paper has reviewed the existing evidence regarding ED and the rising rates of human infertility. Although the number of ED mentioned is not comprehensive, an adequate amount of data has accumulated demonstrating that EDs may have deleterious effects on human reproduction via numerous mechanisms. ED may be blamed for the rising incidence of human reproductive disorders, and may also explain some idiopathic infertility cases, both in men and women.

Endocrine disruption is a serious public health problem that must not be ignored. Authorities should endorse preventive measures regarding exposure to EDs, such as limiting their production in industry worldwide, as the removal of these substances from the environment is neither simple nor cheap.

Meanwhile, the general population might reduce ED exposure by following some simple yet important advice such as i) choose glass over plastics, ii) avoid using plastic containers repeatedly or plastic wrapping to microwave food, iii) reduce consumption of fatty animal products, iv) prefer pesticide-free vegetables and fruits and v) avoid excessive utilisation of cosmetics and other personal care items, particularly during pregnancy. As ED exposure at any dose may impair human development and reproduction, precautionary avoidance of exposure to well-known and putative ED is a prudent attitude.

Further research is needed to improve current knowledge about known ED, and to identify potential endocrine disruptive activity by other chemicals, especially those replacing current ED before they are widely distributed. Dose–effect curves should be thoroughly studied, even at minute concentrations, as all EDs are likely to show non-monotonic responses and low-dose effects, resembling those elicited by endogenous hormones. Also, the impact of exposure to low doses of complex mixtures of ED and the prospective transgenerational effects should be evaluated, specifically concerning genetic polymorphisms, especially during gametogenesis and foetal development. It would be important to examine adult fertility and hormonal parameters of infants inadvertently exposed to contraceptive hormones during pregnancy and of infants fed cow milk/soy-based formula using baby bottles made of different substances, as opposed to breastfed infants. Clinical and laboratorial research on ED is essential, in order to protect wildlife and humans, particularly developing foetuses and children, from permanent effects on fertility.

## Figures and Tables

**Table 1:** Reported agonist and antagonist binding of several ED to ER and AR.

**ED**	**ER agonism**	**ER antagonism**	**AR agonism**	**AR antagonism**
PCB	(33)		(34)	(33)
PBDE	(35)	(35)		(35)
BPA	(36)			(37)
BPS	(38)			
Phthalates	(39)			
Cypermethrin	(40)			
DDT	(36, 40, 41)			(40, 41)
Dieldrin	(40, 41)			(40, 42)
MXC	(36, 40, 41)	(40, 41)		(40, 42)
VCZ	(41)			(40)
DES	(36)			
Phytoestrogens	(36)			

AR, androgen receptor; BPA, bisphenol A; BPS, bisphenol S; DDT, dichlorodiphenyltrichloroethane; DES, diethylstilbestrol; ED, endocrine disruptor; ER, oestrogen receptors; MXC, methoxychlor; PBDE, polybrominated diphenyl ethers; PCB, polychlorinated biphenyls; VCZ, vinclozolin.

**Table 2:** Cellular effects of ED on the testicle.

**Cellular effect**	**ED**
Germ cell apoptosis	Phthalates (112), DES and EE (113)
Reduced steroidogenesis in Leydig cells	PCB (114), phthalates (73), cypermethrin (15), dieldrin (14) and EE (20)

DES, diethylstilbestrol; ED, endocrine disruptor; EE, ethinyl oestradiol; PCB, polychlorinated biphenyls.

**Table 3:** Cellular effects of ED on the ovary.

**Cellular effect**	**ED**
Impaired folliculogenesis	PCB (8), phthalates (166), atrazine (167), MXC (168) and genistein (169)
Follicular atresia	BPA (170)
Meiosis disruption	BPA (170, 171), DES (172) and genistein (173)
Reduced steroidogenesis in granulosa/theca cells	TCDD (174), DDT and MXC (175)

BPA, bisphenol A; DDT, dichlorodiphenyltrichloroethane; DES, diethylstilbestrol; ED, endocrine disruptor; MXC, methoxychlor; PCB, polychlorinated biphenyls; TCDD, 2,3,7,8-tetrachlorodibenzo-*p*-dioxin.
